# Propensity Score-Matched Analysis of Endovascular Treatment and Microsurgery for Unruptured Middle Cerebral Artery Aneurysms: Long-Term Outcomes over 6-Year Follow-Up

**DOI:** 10.3390/jcm15020435

**Published:** 2026-01-06

**Authors:** Lukasz Przepiorka, Katarzyna Przepiórka, Sławomir Kujawski, Karolina Kalinowska, Maria Deczkowska, Tomasz Antczak, Wiktoria Suchcicka, Marcin Skawiński, Andrzej Marchel, Przemysław Kunert

**Affiliations:** 1 Department of Neurosurgery, Medical University of Warsaw, 02-097 Warsaw, Poland; 2 Department of Exercise Physiology and Functional Anatomy, Ludwik Rydygier Collegium Medicum in Bydgoszcz Nicolaus Copernicus University in Toruń, 85-077 Bydgoszcz, Poland

**Keywords:** middle cerebral artery, unruptured aneurysm, propensity score matching, endovascular, microsurgical

## Abstract

**Objectives**: The choice between endovascular treatment and microsurgery for unruptured intracranial aneurysm (UIA) is influenced by aneurysm location, with middle cerebral artery (MCA) UIAs traditionally requiring surgery. This study compares these treatment modalities using propensity score matching (PSM). **Methods**: This single-center analysis included adults with saccular MCA UIAs who underwent treatment. PSM incorporated patient and aneurysm characteristics to create comparable groups. **Results:** Before matching, 124 patients underwent microsurgery and 28 underwent endovascular treatment. With a median follow-up of 76.5 months, 93.4% achieved good functional outcome (modified Rankin Scale [mRS] 0–2), including 117 (93.4%) in the surgical group and 25 (89.3%) in the endovascular group. Complications occurred in 15.3% of surgical and 10.7% of endovascular patients (*p* > 0.05). Three patients developed subarachnoid hemorrhage post-treatment: two from other aneurysms and one from an endovascularly treated MCA UIA. Proximal location predicted worse outcomes (*p* = 0.04), whereas distal location was associated with better outcomes (*p* < 0.01). Ordinal logistic regression revealed no additional associations. After PSM, we did not observe significant between-group differences in complications or mRS at follow-up, and ordinal logistic regression predicting mRS at follow-up revealed no differences. Distal MCA remained associated with better outcomes (*p* < 0.01). No differences in survival were found between groups before or after PSM (log-rank test *p* = 0.34 and *p* = 0.49, respectively). **Conclusions:** No differences in long-term outcomes or complications were observed in this cohort after endovascular treatment vs. microsurgery. Distal location was the only factor associated with favorable outcomes. At a median follow-up of 6.4 years, most patients achieved good functional outcomes. These results likely reflect individualized treatment selection within an experienced team and should be considered exploratory given limited statistical power and generalizability.

## 1. Introduction

The management of unruptured intracranial aneurysms (UIAs) continues to challenge neurosurgeons [1]. With the increasing detectability of UIAs, indications for preventive treatment remain ambiguous in select cases [2,3]. Moreover, selection of an appropriate treatment modality often hinges on the classical dichotomy between microsurgical clipping and endovascular coiling [4]. The choice between these two modalities has traditionally been influenced by the location of the aneurysm. Middle cerebral artery (MCA) UIAs tend to require microsurgical intervention [5,6] due to factors such as wide neck, irregular shape, trifurcations, or branch arteries that might be difficult to detect angiographically [7]. Despite this common tendency, comparisons between endovascular treatment and microsurgery for MCA UIAs remain scarce in the existing literature.

Retrospective comparisons of aneurysms based solely on treatment choice fail to acknowledge inherent differences that affect the treatment choice. This bias can be partially accounted for by matching treatment groups based on their characteristics. In this way, the present study aimed to compare endovascular treatment and microsurgery for MCA UIAs using propensity score matching (PSM) [8].

## 2. Materials and Methods

This retrospective single-center analysis included adults diagnosed with saccular MCA UIA who underwent either microsurgery or endovascular treatment between the years 2009 and 2022, with a minimum available follow-up of 6 months. To allow for direct comparisons between the two groups, we included only patients who underwent treatment of a single saccular MCA UIA. We excluded cases in which more than one aneurysm was treated during the same procedure and all cases with subarachnoid hemorrhage (SAH), even the cases in which other aneurysms were the most probable cause of bleeding. These criteria were used to limit potential bias from complications of different UIA treatments or SAH. The MCA UIA location was categorized as proximal to the bifurcation, at the bifurcation, or distal to the bifurcation [9].

As part of our institutional protocol, we routinely perform posttreatment angiography approximately 6 months after the procedure; in cases of satisfactory occlusion, this serves as the final radiological study. For microsurgical clipping, routine postoperative vascular imaging (angiography, CTA, or MRA) is waived. Since 2018, we have routinely employed intraoperative indocyanine green videoangiography (ICG-VA) to confirm parent artery patency, preservation of branching and perforating vessels, and complete aneurysm obliteration. Prior to this, early postoperative CTA was selectively performed only in cases of intraoperative uncertainty [10].

### Propensity Score Matching and Statistical Analysis

PSM was used to account for potential confounding variables and to create comparable groups for the analysis. This matching was performed using a generalized linear model based on distance, employing the nearest-neighbor matching method with a 1:1 ratio. The methodological approach utilized the “MatchIt” package [11].

The process incorporated the following covariates: age, sex, aneurysm dome size, aneurysm neck size, hypertension, earlier SAH from another aneurysm (i.e., past medical history significant for previous aneurysmal rupture from a different aneurysm), and MCA location (proximal, bifurcation, distal) [11]. The “Optimal” method was chosen. Missing data were imputed using the median value. The MASS package was used to fit ordinal logistic regression models for ordinal outcomes as the modified Rankin Scale (mRS) [12]. The sjPlot, broom, and ggplot2 packages were utilized to create publication-ready tables and plots of model results [13,14,15]. Survival analysis (in terms of years) was performed using the survival and survminer packages [16,17]. The Wald chi-squared test in the coxphw package was used when the assumption of proportionality of hazards was not met [18]. Aspect ratios were compared across treatment strategies using the Kruskal–Wallis test. Post hoc pairwise comparisons were explored using Dunn-type rank-based tests with Holm adjustment for multiple comparisons.

## 3. Results

### 3.1. Group Description Prior to Matching

A total of 124 patients (91 females, 33 males) with a median age of 59.5 (interquartile range [IQR] 12) years underwent microsurgery, and 28 patients (22 females, 6 males) with a median age of 58 (IQR 14.25) years underwent endovascular treatment. The median total number of aneurysms was 1 in both groups, with a range of 1–6 in the microsurgery group and 1–5 in the endovascular treatment group.

#### 3.1.1. Surgical and Endovascular Techniques

The microsurgery group consisted of patients undergoing craniotomy with clip reconstruction, with the exception of a 74-year-old man whose surgery included rapid ventricular pacing, which was subsequently complicated by Takotsubo cardiomyopathy. He had a 16 mm aneurysm but otherwise tolerated the procedure fairly well. The microsurgery group also included one patient who had previously received unsuccessful endovascular treatment.

In the endovascular treatment group, coiling alone was used in 14 cases (50%), balloon-assisted coiling in 10 cases (35.7%), stent-assisted coiling in 2 cases (7.1%), and a flow diverter without additional coiling in 2 cases (7.1%). None of the patients underwent treatment with intrasaccular devices. Median aneurysm aspect ratios were similar among coiling-based techniques, including coiling alone (median 1.54, IQR 1.43–2.00), balloon-assisted coiling (median 1.60, IQR 1.19–1.94), and stent-assisted coiling (median 1.56, IQR 1.45–1.68), whereas aneurysms treated with flow diversion demonstrated higher aspect ratios (median 6.59, IQR 4.75–8.42). Aspect ratios did not differ significantly across treatment groups (Kruskal–Wallis H = 5.69, *p* = 0.128). Although aneurysms treated with flow diversion demonstrated higher median aspect ratios, no pairwise comparisons reached statistical significance after adjustment for multiple testing.

#### 3.1.2. Functional Outcomes, Complications, and Survival

Prior to treatment, most of the patients (*n* = 142; 93.4%) were in good health (mRS = 0), 5 patients (3.3%) had an mRS score of 1, 3 patients (2.1%) had an mRS score of 2, and 1 patient (1.4%) had moderate disability (mRS = 3). The median follow-up was 76.5 months (Q1: 37, Q3: 112), at which point 93.4% of patients had a good functional outcome (mRS = 0: 129 patients, 84.9%; mRS = 1: 9 patients, 5.9%; mRS = 2: 4 patients, 2.6%). Accordingly, good outcomes were achieved in both the microsurgery (93.4%, 117/124) and endovascular treatment groups (89.3%, 25/28). The remaining patients exhibited poor functional outcomes (mRS = 3, 4 patients, 2.6%; mRS = 4, 4 patients, 2.6%; mRS = 5, 0 patients; mRS = 6, 2 patients, 1.3%). Seventeen patients (10.5% of the whole group) had a worse mRS score in the long-term follow-up, of which 9 (5.9%) were directly related to the MCA UIA treatment.

Overall, 15.3% of patients in the microsurgery group experienced complications (*n* = 9 ischemic strokes, *n* = 3 hemorrhagic strokes, *n* = 2 requiring surgical removal, *n* = 3 intracranial hypotension, *n* = 1 Takotsubo syndrome with intracranial hypotension, *n* = 1 poor wound healing, *n* = 1 CSF leak, *n* = 1 epileptic seizures and psychotic episode, *n* = 1 exacerbation of heart failure) and 10.7% in the endovascular treatment group (*n* = 1 ischemic stroke, *n* = 1 in-stent thrombosis during the procedure, *n* = 1 in-stent thrombosis after scheduled discontinuation of dual antiplatelet therapy). When restricted to procedure-related events, these occurred in 14.5% of patients in the microsurgery group (18/124) and 7.1% in the endovascular treatment group (2/28); systemic events such as exacerbation of heart failure and in-stent thrombosis occurring after planned discontinuation of dual antiplatelet therapy were not considered directly procedure-related.

Three patients suffered from SAH following treatment: two from different aneurysms and one from a treated MCA UIA. A 58-year-old female with multiple UIAs, including a 5 mm right MCA UIA, was treated with coiling, and her latest 1.5-year radiological follow-up revealed no recanalization. Unfortunately, she experienced a hemorrhage and died 11 years after treatment of the MCA UIA.

**Figure 1:**
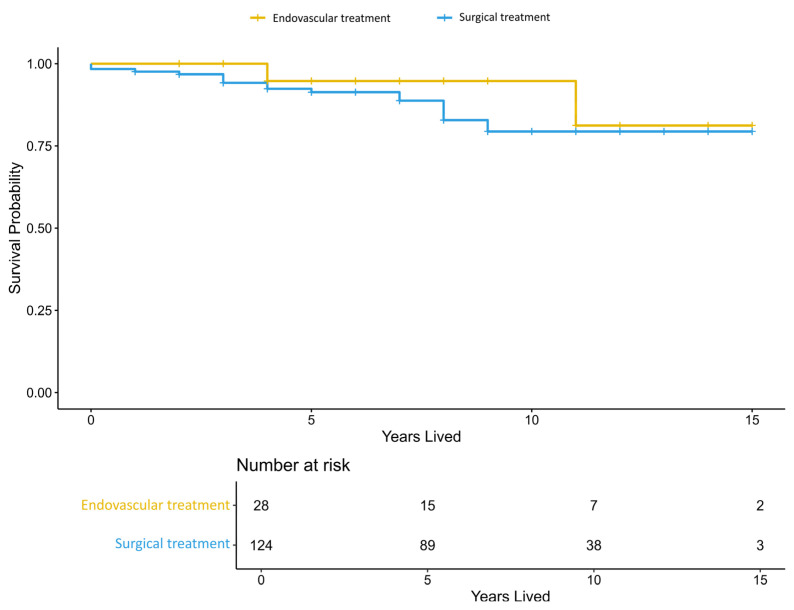
Kaplan–Meier curves comparing survival over time (measured in years lived) before propensity score matching between the microsurgery (blue) and endovascular treatment (orange) groups.

We obtained additional data from national healthcare records regarding the date of death for deceased patients or confirmation that the patient was alive. The Kaplan–Meier survival curves are presented in Figure 1. We did not find a significant difference in survival between the endovascular treatment and microsurgery groups (Wald chi-squared *p* = 0.34). Eighteen deaths occurred in the microsurgery group and two deaths in the endovascular treatment group

#### 3.1.3. Pre-Matching Statistical Evaluation of Functional Outcomes

Before PSM, we observed no significant differences between the two treatment groups in terms of the mRS score at follow-up (Table 1). In the whole study group, the proximal MCA UIA location had a significant odds ratio (OR) for worse mRS score at follow-up (OR = 5.56, 95% confidence interval [CI] 1.05–29.49, *p* = 0.04). In contrast, distal MCA UIA had a significant OR for better mRS score at follow-up (*p* < 0.001; Table 1); however, this finding likely reflects model separation. The ordinal logistic regression model predicting mRS score at follow-up without controlling for confounding factors did not reveal any significant differences (Table S1). We also observed no significant differences in the occurrence of treatment complications between the endovascular treatment and microsurgery groups (Table 2).

#### 3.1.4. Pre-Matching Radiological Follow-Up in the Endovascular Group and Retreatment Rates

Follow-up radiological data were available for 28 of 29 patients (96.6%) in the endovascular treatment group. Two patients (7.1%) underwent additional interventions: one required frontotemporal craniotomy for coil removal followed by microsurgical clipping of the aneurysm neck, and the other received two further coiling sessions, the second with balloon assistance. In the remaining 26 patients, angiography demonstrated complete occlusion in all but one case, which showed a small stable residual neck remnant. None of the patients in the surgical group underwent additional treatment.

### 3.2. Group Description After Matching

**Figure 2:**
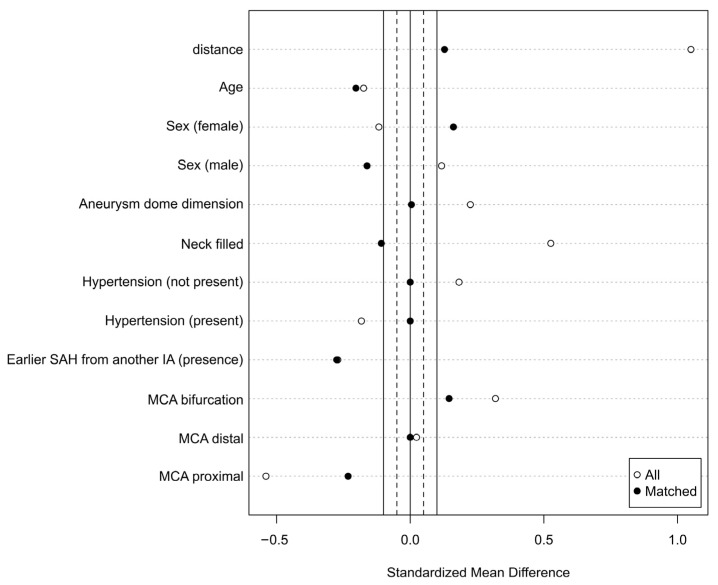
Standardized mean difference between all patients and matched patients.

The PSM process incorporated the following covariates: age, sex, aneurysm dome size, aneurysm neck size, hypertension, earlier SAH from another aneurysm, and MCA location (bifurcation, distal, proximal) (Figure 2).

After matching, there were 28 patients in both treatment groups (24 females in the microsurgery group and 22 females in the endovascular treatment group). The mean age ± SD was 57.9 ± 10.8 and 60.1 ± 9.3 in the microsurgery and endovascular treatment group, respectively. The median total number of intracranial aneurysms in a patient was 1 in the microsurgery group (range 1–3) and 1.5 in the endovascular treatment group (range 1–5). We observed no significant differences between the endovascular treatment and microsurgery groups in terms of the mRS score at follow-up (Table 3). Similarly, ordinal logistic regression models predicting mRS score at follow-up without controlling for confounding factors revealed no significant differences (Table S2). Distal locations had a significant OR for better mRS score at follow-up (*p* < 0.001; Table 3); however, the estimated OR of zero suggests model separation and should be interpreted with caution.

After PSM, we observed no significant differences in the occurrence of treatment complications between the endovascular treatment and microsurgery groups (Table 4). The model did not contain “earlier SAH from another UIA”, as this variable contained too few observations to serve as a meaningful predictor (2 occurrences in the microsurgery group and 4 in the endovascular treatment group).

**Figure 3:**
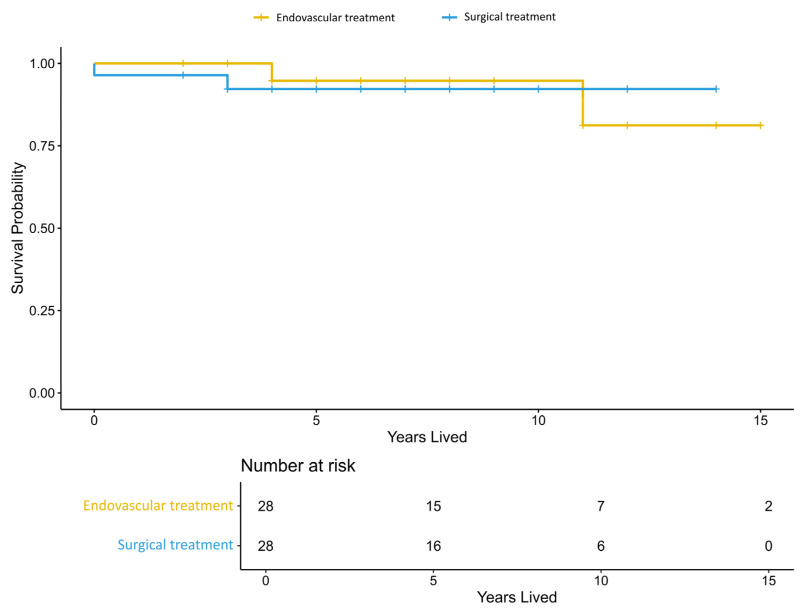
Kaplan–Meier curves showing survival over time (measured in years lived) after propensity score matching. The endovascular treatment group is shown in orange, and the microsurgery group is shown in blue.

Figure 3 shows the Kaplan–Meier survival curves after PSM. No significant differences in survival were observed between the endovascular treatment and microsurgery groups (log-rank test *p* = 0.49). A total of two deaths were observed in the microsurgery group and two deaths in the endovascular treatment group.

## 4. Discussion

### 4.1. Main Findings

In this study at a single center offering multidisciplinary treatment modalities for MCA UIA, the long-term results of matched patients are not significantly different in terms of the mRS score. Even prior to matching our cases, there were no significant differences in functional outcomes between the two treatment groups.

Similar to our findings, Padmanaban et al. found similar safety of microsurgery and endovascular treatment for wide-neck MCA aneurysms [19]. However, follow-up was available for only 52.9% of the patients in their study, whereas we excluded only 10% (17/169) of all MCA UIAs treated in the selected time period due to lack of follow-up. Moreover, in Padamanaban et al.’s study, the length of follow-up was 404 and 157.5 days in the endovascular treatment and microsurgery group, respectively. In the present study, the median follow up was 56.5 (Q1: 25.75, Q3: 116) and 79 (Q1: 40, Q3: 112) months in the endovascular treatment and microsurgery group, respectively.

We also analyzed the risk of hemorrhage from treated MCA aneurysms. There was one case of hemorrhage from an endovascularly treated MCA UIA, which resulted in the death of the patient. Nonetheless, microsurgery was characterized by a higher percentage of complications overall (15.2% vs. 9.7%), of which ischemic stroke was the most common (10/22, 45.4% of all surgical complications). Despite these complications, in the long-term follow-up, 89% (138/154) of patients achieved good functional outcomes (mRS = 0–1) and 94.1% did not experience worsening in terms of the mRS score.

### 4.2. Microsurgery and Endovascular Treatment

In 2013, Rodriques-Hernandez et al. reported good clinical outcomes (defined as mRS scores of 0–2) of microsurgery in 92.0% of patients with MCA UIAs [20], which is comparable with our results of good functional outcomes in 93.4% of the microsurgery group. They concluded that microsurgery is the treatment of choice for MCA aneurysms as it offered complete obliteration in 98.3% of unruptured cases [20]. Notably, their study served as a beacon for microsurgery because its publication followed the major shift towards endovascular treatment of intracranial aneurysms after the International Subarachnoid Aneurysms Trial (ISAT) [21].

Yang et al. recently reported a series of 92 surgically treated ruptured and unruptured MCA aneurysms with good and durable outcomes [22]. Similarly, in our cohort, surgical treatment was durable, with no patient requiring retreatment. Yang et al. also provided a review of rates of complications after endovascular treatment of MCA aneurysms, which ranged from 5.1% based on the Mayo Clinic’s experience with 36 MCA aneurysms [23] to 25.5% based on 59 aneurysms reported by Quadros et al. [24]. Importantly, since the latter publication in 2007, vast improvements have been made in endovascular treatment. Consistent with this trend, the complication rate in the endovascular treatment group in the present study was 10.7%.

In 2011, prior to the introduction of intrasaccular devices, Brinjikji et al. postulated that, “MCA location alone is not a per se contraindication for endovascular coil embolization.” [23]. This aligns with our findings, as the majority of patients underwent coiling (24/28, 85.7%), demonstrating that proper endovascular treatment in appropriately selected patients can yield favorable results, as 89.3% of patients achieved good outcomes. Importantly, Brinjikji et al. also highlighted a limitation of endovascular treatment, reporting a 9.2% incidence of minor recurrences that did not require treatment and a 9.6% incidence of major recurrences requiring re-treatment, which is in line with the 7.1% retreatment rate observed in our series [23]. A 2021 multicenter study by Diestro et al. on the use of flow diverters for predominantly unruptured MCA aneurysms suggested that this may be a viable treatment option. However, outcomes were less favorable than those reported in the surgical literature [25].

### 4.3. Complications

In one of the largest contemporary series of microsurgically treated MCA UIAs, treatment-related complications occurred in 2.8% of patients (20/716 patients with 750 aneurysms) [26]. In addition, Yang et al. reported new postoperative neurological morbidity in 15.2% of patients (7/46 in the unruptured cohort) with MCA UIAs [22]. Other available studies reported overall lower or similar complication rates, but our study’s significance and differences lie in the inclusion of not only MCA UIAs at a bifurcation, but also other locations, namely the M1 segment proximal to the bifurcation and all distally located aneurysms. Heros and Fritsch reported that locations other than the bifurcation may comprise up to 19% of all MCA UIAs; in our population it was 7.9% [27,28].

Although some studies define treatment complications based on criteria such as “worsening of the mRS,” “new neurological deficit,” or “treatment-related” events only, we chose a broader approach. To better reflect patient-oriented outcomes rather than surgery-centered definitions, we reported all complications regardless of their attribution. This approach emphasizes the overall patient experience rather than limiting the analysis to complications directly linked to the surgical procedure.

### 4.4. Summary

A 2015 systematic review and meta-analysis recommended clipping over coiling for MCA UIAs [29]. This position was reaffirmed by an updated 2018 meta-analysis, which also noted advances in endovascular techniques [30]. These developments in endovascular devices have expanded the range of MCA UIAs amenable to endovascular treatment with good results. Changes in the MCA UIA treatment landscape were reflected by our institutional management; although microsurgery remained an adequate consideration for most MCA UIAs due to their common characteristics, such as wide neck and bifurcation location, an increasing number of patients have undergone endovascular treatment over time. This has resulted in good long-term results.

### 4.5. Limitations and Future Studies

This study is limited by its retrospective, single-center nature and relatively small sample size, particularly the low number of poor-outcome events. Therefore, multivariable analyses, including logistic regression and PSM, are underpowered and prone to model instability, wide confidence intervals, and separation, which limits the reliability of effect estimates and substantially restricts external validity. Similarly, the survival analysis is underpowered by the small endovascular sample, which limits the robustness of long-term outcome estimates.

Although we attempted to limit bias related to baseline group differences by using PSM, this approach remains far from the gold standard of a randomized controlled trial and cannot account for unmeasured confounders. In particular, operator expertise, institutional experience, complication management, and shared decision making in a multidisciplinary setting likely influenced both treatment selection and outcomes, especially in a high-volume referral center.

In addition, our endovascular treatment group consisted of patients treated with a variety of techniques reflecting real-life scenarios in which the neurointerventionalist chooses the most appropriate treatment for the angioarchitecture of the aneurysm. While this enhances clinical relevance, it introduces additional heterogeneity that may further confound comparisons between treatment modalities.

Furthermore, future studies should include intrasaccular devices, which are changing the landscape of aneurysm treatment, particularly of MCA UIAs [31]. Finally, it is important to incorporate patient-centered metrics into clinical neurosurgical research and to place more emphasis on patients’ quality of life following treatment.

## 5. Conclusions

In this homogeneous group of MCA UIAs treated at a single referral center in a multidisciplinary setting, there were no significant differences between endovascular treatment and microsurgery in terms of functional outcome and complications during a long-term follow-up, which was also apparent after PSM. A distal location of the MCA UIA emerged as the only factor in our analysis with a significant OR for better mRS score; however, this finding should be interpreted with caution given the limited sample size. At the median follow-up of 6.4 years, the majority of patients achieved good functional outcomes. Importantly, these results likely reflect appropriate, individualized treatment selection within an experienced multidisciplinary team. Given the limited statistical power, the findings should be considered exploratory, with limited generalizability beyond similar institutional settings.

## Figures and Tables

**Table 1:** Ordinal logistic regression model predicting the modified Rankin Scale at follow-up in all subjects (*n* = 124 microsurgery, *n* = 28 endovascular treatment) while controlling for confounding factors.

Predictors	Odds Ratios	CI	*p*
Surgical treatment	0.53	0.15–1.84	0.311
Age (years)	1.04	0.99–1.09	0.150
Sex (male)	1.65	0.56–4.88	0.361
Aneurysm dome dimension (mm)	1.06	0.98–1.14	0.153
Neck size (mm)	1.15	0.84–1.59	0.371
Hypertension (present)	1.01	0.34–2.98	0.984
Earlier SAH from another aneurysm	2.69	0.59–12.29	0.201
MCA UIA location: distal	0.00	0.00–0.00	**<0.001**
MCA UIA location: proximal	5.56	1.05–29.49	**0.044**
Observations	152		
R^2^ Nagelkerke	0.140		

Confounding factors were age, sex, aneurysm dome size, aneurysm neck size, hypertension, earlier subarachnoid hemorrhage (SAH) from another aneurysm, and middle cerebral artery (MCA) location (proximal, bifurcation, distal). The MCA bifurcation location category served as a reference. mRS, modified Rankin Scale.

**Table 2:** Binomial logistic regression model predicting the occurrence of treatment complications in all subjects (*n* = 124 microsurgery, *n* = 28 endovascular treatment) while controlling for confounding factors.

Predictors	Odds Ratios	CI	*p*
Surgical treatment	2.04	0.54–10.97	0.344
Age (years)	1.02	0.98–1.08	0.336
Sex (male)	1.79	0.62–4.89	0.266
Aneurysm dome dimension (mm)	1.05	0.96–1.15	0.246
Neck size (mm)	0.94	0.66–1.33	0.726
Hypertension (present)	1.07	0.40–3.06	0.894
Earlier SAH from another aneurysm	1.62	0.22–7.56	0.573
MCA UIA location: distal	1.47	0.07–11.11	0.744
MCA UIA location: proximal	4.88	0.59–32.73	0.104
Observations	152		
R^2^ Nagelkerke	0.140		

Confounding factors were age, sex, aneurysm dome size, aneurysm neck size, hypertension, earlier subarachnoid hemorrhage (SAH) from another aneurysm, and middle cerebral artery (MCA) location (proximal, bifurcation, distal). The MCA bifurcation location category served as a reference.

**Table 3:** Ordinal logistic regression model predicting the modified Rankin Scale at follow-up in subjects after propensity score matching (*n* = 28 microsurgery, *n* = 28 endovascular treatment) while controlling for confounding factors.

Predictors	Odds Ratios	CI	*p*
Surgical treatment	0.30	0.05–1.82	0.185
Age (years)	1.03	0.95–1.13	0.416
Sex (male)	0.83	0.06–10.77	0.881
Aneurysm dome dimension (mm)	1.08	0.96–1.20	0.197
Neck size (mm)	0.86	0.41–1.81	0.687
Hypertension (present)	0.90	0.13–6.34	0.915
Earlier SAH from another aneurysm	1.37	0.10–19.73	0.813
MCA UIA location: distal	0.00	0.00–0.00	**<0.001**
MCA UIA location: proximal	2.49	0.30–20.84	0.391
Observations	56		
R^2^ Nagelkerke	0.163		

Confounding factors were presence of hypertension, age, aneurysm dome dimensions, previous subarachnoid hemorrhage from another intracranial aneurysm, aneurysm location, and aneurysm neck dimension. Left middle cerebral artery bifurcation aneurysm category location served as a reference. mRS, modified Rankin Scale.

**Table 4:** Binomial logistic regression model predicting the occurrence of treatment complications in subjects after propensity score matching (*n* = 28 microsurgery, *n* = 28 endovascular treatment) while controlling for confounding factors.

Predictors	Odds Ratios	CI	*p*
Surgical treatment	1.31	0.15–12.44	0.802
Age (years)	1.01	0.91–1.15	0.804
Sex (male)	3.36	0.17–52.58	0.366
Aneurysm dome dimension (mm)	1.15	1.00–1.51	0.143
Neck size (mm)	0.78	0.28–1.72	0.592
Hypertension (present)	1.06	0.09–25.65	0.963
Earlier SAH from another aneurysm	19.63	0.38–1491.69	0.127
MCA UIA location: distal	5.36	0.21–83.52	0.226
Surgical treatment	1.31	0.15–12.44	0.802
Observations	152		
R^2^ Nagelkerke	0.140		

Confounding factors were age, sex, aneurysm dome size, aneurysm neck size, hypertension, earlier subarachnoid hemorrhage (SAH) from another aneurysm, and middle cerebral artery (MCA) location (proximal, bifurcation, distal). The MCA bifurcation location category served as a reference.

## Data Availability

The data presented in this study are available on request from the corresponding author after acceptance of all the co-authors.

## Supplementary Materials

The following supporting information can be downloaded at: https://www.mdpi.com/article/10.3390/jcm15020435/s1, Table S1: Ordinal logistic regression model predicting the modified Rankin Scale at follow-up in all subjects (*n* = 124 microsurgery, *n* = 28 endovascular treatment) without controlling for confounding factors; Table S2: Ordinal logistic regression model predicting the modified Rankin Scale at follow-up after propensity score matching (*n* = 28 microsurgery, *n* = 28 endovascular treatment) without controlling for confounding factors.

## Abbreviations

The following abbreviations are used in this manuscript:

IQR	interquartile range
MCA	middle cerebral artery
OR	odds ration
PSM	propensity score matching
SAH	subarachnoid hemorrhage
UIA	unruptured intracranial aneurysms
