# Supplementary material for: Propensity Score-Matched Analysis of Endovascular Treatment and Microsurgery for Unruptured Middle Cerebral Artery Aneurysms: Long-Term Outcomes over 6-Year Follow-Up
Source: J Clin Med. 2026 Jan 6;15(2):435. doi: 10.3390/jcm15020435 (PMC12841974; doi:10.3390/jcm15020435)
Supplement: Supplementary file 1 [file jcm-15-00435-s001.zip › Table S1.pdf]

Table S1. Ordinal logistic regression model predicting the modified Rankin Scale at follow-up in all subjects (n=124 microsurgery, n=28 endovascular treatment) without controlling for confounding factors.

	mRS at follow-up		
Predictors	*Odds Ratios*	*CI*	*p*
0|1	3.08	1.31 – 7.22	**0.010**
1|2	5.46	2.21 – 13.46	**<0.001**
2|3	7.89	3.03 – 20.57	**<0.001**
3|4	13.54	4.58 – 40.01	**<0.001**
4|6	41.71	8.65 – 201.14	**<0.001**
Surgical treatment	0.46	0.17 – 1.25	0.126
Observations	152		
R^2 Nagelkerke	0.020		

mRS – modified Rankin scale
