# Supplementary material for: Propensity Score-Matched Analysis of Endovascular Treatment and Microsurgery for Unruptured Middle Cerebral Artery Aneurysms: Long-Term Outcomes over 6-Year Follow-Up
Source: J Clin Med. 2026 Jan 6;15(2):435. doi: 10.3390/jcm15020435 (PMC12841974; doi:10.3390/jcm15020435)
Supplement: Supplementary file 1 [file jcm-15-00435-s001.zip › Table S2.pdf]

Table S2. Ordinal logistic regression model predicting the modified Rankin Scale at follow-up after propensity score matching (n=28 microsurgery, n=28 endovascular treatment) without controlling for confounding factors.

	**mRS at follow-up**		
Predictors	*Odds Ratios*	*CI*	*p*
0|1	3.11	1.30 – 7.43	**0.012**
1|2	5.07	1.91 – 13.47	**0.002**
2|3	8.01	2.59 – 24.78	**0.001**
3|4	16.83	3.73 – 75.91	**<0.001**
4|6	16.83	3.73 – 75.91	**<0.001**
Surgical treatment	0.25	0.04 – 1.36	0.106
Observations	56		
R^2 Nagelkerke	0.075		

mRS – modified Rankin scale
